# Clinical Efficacy of Deep Transcranial Magnetic Stimulation in Psychiatric and Cognitive Disorders: Protocol for a Systematic Review

**DOI:** 10.2196/45213

**Published:** 2023-05-26

**Authors:** Anne-Marie Di Passa, Melissa Dabir, Allan Fein, Saba Khoshroo, Carly McIntyre-Wood, Emma Marsden, Emily MacKillop, Jane De Jesus, James MacKillop, Dante Duarte

**Affiliations:** 1 Department of Psychiatry and Behavioural Neurosciences McMaster University Hamilton, ON Canada; 2 Peter Boris Centre for Addictions Research St Joseph's Healthcare Hamilton Hamilton, ON Canada; 3 Michael G DeGroote Centre for Medicinal Cannabis Research McMaster University Hamilton, ON Canada

**Keywords:** cognitive disorders, deep rTMS, deep TMS, deep transcranial magnetic stimulation, dTMS, H-coil, prospective meta-analysis, psychiatry, systematic review protocol

## Abstract

**Background:**

Transcranial magnetic stimulation (TMS) is a widely used noninvasive brain stimulation technique for psychiatric and cognitive disorders. In recent years, deep TMS (dTMS) has shown promise as an enhanced form of TMS able to stimulate deeper brain structures and target broader networks. Various magnetic Hesed-coil (H-coil) designs—a novel feature of dTMS—have been used to stimulate brain regions implicated in the pathophysiology of specific psychiatric and cognitive disorders, thereby producing therapeutic effects. Given the novelty of dTMS in psychiatry, little is known about the clinical efficacy of dTMS across psychiatric and cognitive disorders—that is, whether dTMS performs superiorly to sham or control.

**Objective:**

In this paper, we outline a protocol for a systematic review investigating the clinical efficacy of dTMS. The primary objective is to conduct a systematic review of the literature on dTMS for psychiatric and cognitive disorders and, if feasible, a meta-analysis to compare the efficacy of active dTMS versus sham/control for psychiatric disorders. Dementia and related cognitive disorders will also be examined. A secondary objective will be to examine subgroup differences (by age, sex, H-coil design, and dTMS parameters [ie, pulses per session, percentage of motor threshold, etc]) to evaluate whether dTMS differentially influences clinical outcomes based on these factors.

**Methods:**

A comprehensive search of the APA PsycINFO, Embase, MEDLINE, and PubMed databases will be conducted using keywords such as “H-coil” and “dTMS.” Two authors (AD and MD) will be responsible for screening relevant articles, assessing article eligibility (according to predetermined inclusion and exclusion criteria), and data extraction. All included articles will undergo a quality and risk of bias assessment. Data from included articles will be summarized qualitatively in a systematic review. If a sufficient number of equivalent studies are available, a meta-analysis will be performed to (1) determine the effect of active versus sham dTMS (or another control arm) across psychiatric and cognitive disorders, and (2) examine subgroup effects of clinical outcomes.

**Results:**

The preliminary search rendered a total of 1134 articles from the APA PsycINFO, Embase, and MEDLINE databases. After full-text screening, 21 eligible articles remained. One additional article was identified from the references section of an existing systematic review. In total, 22 eligible articles were included. Data extraction and quality of assessment procedures are ongoing.

**Conclusions:**

We will outline the evidence relating to the clinical efficacy of dTMS in various psychiatric and cognitive disorders. The results of the prospective systematic review will provide clinicians with valuable insight into the clinical (ie, participant age, sex, psychiatric or cognitive disorder, etc) and methodological factors (ie, H-coil design, dTMS parameters, etc) which may contribute to dTMS efficacy, and thereby may assist clinicians in their decision to prescribe dTMS for specific psychiatric and cognitive disorders.

**Trial Registration:**

PROSPERO CRD42022360066; https://tinyurl.com/5ev6byrn

**International Registered Report Identifier (IRRID):**

DERR1-10.2196/45213

## Introduction

Psychiatric [1,2] and cognitive disorders [3] are leading causes of disability and burden worldwide and are associated with substantially poorer quality of life [4-7]. From 1990 to 2019, the number of disability-adjusted life-years due to psychiatric disorders has increased globally from 80.8 million to 125.3 million. [2] Of greater concern, it is estimated that 20%-60% of patients with psychiatric disorders are treatment-resistant to first-line medications [8]. In particular, treatment resistance occurs in approximately one third of patients with major depressive disorder [9,10], 30%-60% of patients with schizophrenia [11-13], and 40%-60% of patients with obsessive-compulsive disorder [14,15]. As for cognitive disorders, dementia is on the rise as a leading cause of global mortality [16]. Further, older adults with mild cognitive impairment show an increased risk of mortality [17,18]. Currently, there are no effective treatment options to delay the progression of mild cognitive impairment and dementia [19], nor are there effective interventions to manage the behavioral and psychological burdens of such conditions [20-23].

Given the prevalence of treatment resistance in response to first-line pharmacological interventions in psychiatric disorders, as well as the lack of available treatment options to effectively treat and manage cognitive disorders, new effective treatments are urgently needed. One such technique is brain stimulation.

Brain stimulation methods have shown increasing promise and widespread acceptance as nonpharmacological interventions to treat various psychiatric [24,25] and cognitive disorders [26,27]. Electroconvulsive therapy [28] and transcranial magnetic stimulation (TMS) [29,30] are the most commonly used treatments, which involve the stimulation of the brain through electrical currents and magnetic pulses, respectively. Traditional single-pulse TMS is used to explore brain functioning, whereas repetitive TMS (rTMS) is administered to induce brain activity that lasts beyond the stimulation period [31]. These noninvasive stimulation techniques offer potentially propitious therapy for neurological and psychiatric disorders [32]. Research on other therapy options, such as vagus nerve stimulation [33,34] and deep brain stimulation [35-37], have continued to accumulate, offering further insight into these treatment options for psychiatric disorders.

Another promising method—known as deep TMS (dTMS)—is a noninvasive technique allowing for stimulation of deeper cortical areas and presumably deeper neural networks [38,39]. Since the approval of dTMS in 2013 by the US Food and Drug Administration for major depressive disorder [40], this method of intervention has gained popularity for the treatment of diverse psychiatric disorders.

The aim of dTMS is to modulate brain activity by applying an electrical current over the scalp. Brief magnetic pulses cause widespread neural depolarization in deep regions of the brain, resulting in reduced focal distribution of the electric field [41]. Trains of pulses can be delivered at high-frequency stimulation (>5 Hz) to increase neuronal excitability or at low-frequency stimulation (~1 Hz) to reduce neural excitability [42]. The use of a Hesed-coil (H-coil) dTMS system is a relatively novel noninvasive method that stimulates deeper regions of the brain in comparison to traditional rTMS [43,44]. While traditional TMS figure-8 coils generally stimulate subdural cortical targets up to 0.7 cm [44], dTMS target areas are up to 4 cm beneath the surface depending on the chosen H-coil [45]. More than 20 different types of H-coils have been developed to effectively stimulate specific brain structures [46]. The H-coil configuration received clearance from the US Food and Drug Administration for the treatment of major depression (H1-coil), obsessive-compulsive disorder (H7-coil), and smoking cessation (H4-coil), and anxious depression, in 2013, 2018, 2020, and 2021, respectively. Various H-coil designs have been shown to be safe and efficacious in the treatment of various neuropsychiatric conditions [47-49].

H-coils have complex winding designs and broad dimensions to encompass a larger surface area for stimulation of the electric field [46]. The different types of H-coil designs reflect the subdural depth and volume of stimulation, as well as the region of stimulation. The H1-coil was designed to stimulate the bilateral prefrontal cortex with preference to the left dorsolateral prefrontal cortex of approximately 1.8 cm subdurally [50]. The volume of stimulation for the H1-coil configuration typically encompasses 18 cm^3^ [51]. Further, the H7-coil design primarily stimulates 3 cm of subdural depth and 40.3 cm^3^ of breadth in the medial prefrontal cortex and anterior cingulate cortex [51], while the H4-coil targets the bilateral insula and ventrolateral and dorsolateral prefrontal cortex with a depth of 1.5 cm and a volume of 15.2 cm^3^ [45]. In comparison, the traditional figure-8 coil used in rTMS stimulates 0.7 cm below the scalp, encompassing a volume of 3 cm^3^ [51]. The most commonly used dTMS protocol involves high-frequency (18-20 Hz) and high-intensity (120% of the resting motor threshold) stimulation delivered for 20 days [41].

Given the increasing interest in the application of dTMS in psychiatry [52], it is critical that its clinical effects are systematically appraised. As dTMS is a relatively novel form of therapy for psychiatric and cognitive disorders, assessments of its clinical efficacy are limited within the empirical body of literature.

A single systematic review and meta-analysis have examined the clinical efficacy of dTMS for treatment-resistant depression [52]. The authors highlight dTMS as an effective intervention, especially when administered in conjunction with antidepressants. However, systematic reviews examining the clinical efficacy of dTMS for other psychiatric conditions remain sparse. One systematic review [53] explored the effects of rTMS in patients with posttraumatic stress disorder. However, only 1 sham-controlled dTMS study [49] was included in this review. Another systematic review [54] examined the effects of dTMS for substance use disorders; however, clinical efficacy was not appraised due to the limited number of sham-controlled trials.

To date, no systematic review has investigated the clinical efficacy of dTMS across multiple psychiatric and cognitive disorders, as determined by the examination of sham-controlled or controlled (without sham coil) trials. There is a growing need for a new systematic review to synthesize the findings of recent dTMS clinical trials in patients with various psychiatric and cognitive disorders, including Alzheimer disease [55], attention-deficit or hyperactivity disorder [56], obsessive-compulsive disorder [57], posttraumatic stress disorder [58], and substance use disorder [59]. Thus, the objective of the proposed systematic review and prospective meta-analysis is to compare the efficacy of dTMS active treatment versus sham stimulation (or another type of control treatment) across psychiatric disorders and pertinent cognitive disorders.

Specifically, the proposed systematic review that follows this paper’s protocol will aim to:

1. Systematically review and summarize existing research to examine the clinical efficacy of dTMS versus sham/controls for psychiatric disorders and (if available) cognitive disorders.
2. Integrate statistical results of included clinical trials in a meta-analysis, if feasible (ie, adequate numbers of substantively equivalent studies are present), to determine the effect of dTMS active stimulation versus sham (or control arm) across psychiatric and pertinent cognitive disorders.
3. Conduct subgroup analyses (by age, gender, coil design, and dTMS parameter) to determine if dTMS differentially influences clinical outcomes within these subgroups.

## Methods

### Search Strategy

A systematic search of the PubMed, APA PsycINFO, Embase, and MEDLINE databases will be conducted to identify relevant articles. The Cochrane database will also be explored for pertinent articles. Two authors (AD and MD) will be responsible for independently conducting the searches. The core keywords used in the searches will include “dTMS,” “deep rTMS,” “deep TMS,” “deep transcranial magnetic stimulation,” and “H-coil.” Table 1 provides a summary of the search terms that will be used for each database. Automatic filters will be applied to limit search results to clinical trials and randomized controlled trials. No date limits will be applied. Moreover, a manual search of existing meta-analyses and systematic reviews on dTMS for psychiatric and cognitive disorders will be conducted to identify other potential articles from the reference section.

**Table 1:** Search strategies used to identify relevant articles from databases. Searches will be limited to clinical trials and randomized controlled trials written in English. No date limits will be applied.

Databases	Search strategy	Final search terms
PubMed, Cochrane, and OVID (APA PsycINFO, Embase, and MEDLINE)	dTMS “deep TMS” “deep transcranial magnetic stimulation” “deep rTMS” “deepTMS” “H-coil*” “H coil*”	1 or 2 or 3 or 4 or 5 or 6 or 7

### Study Selection: Inclusion and Exclusion Criteria

All articles will be independently screened and assessed for eligibility by 2 authors (AD and MD) according to the predetermined inclusion criteria. We will include clinical trials reporting the clinical effect of dTMS interventions for psychiatric and cognitive disorders compared to a sham-controlled or control arm. The full inclusion and exclusion criteria are presented in Textbox 1.

**Inclusion and exclusion criteria.**

**Inclusion criteria:**

- Article type
  Clinical trials and randomized controlled trials
  Studies including a sham or control condition
  Studies comparing active deep transcranial magnetic stimulation to a sham or control condition
  Articles of any publication year
- Study participants
  Participants with a valid diagnosis of a psychiatric or cognitive disorder according to a recognized diagnostic manual (ie, the Diagnostic and Statistical Manual of Mental Disorders, Fourth or Fifth edition [DSM-IV, DSM-5], the International Classification of Diseases, Eleventh edition [ICD-11]), or a clinician
- Language
  Articles written in English

**Exclusion criteria:**

- Article type
  Conference abstracts
  Letters to the editor
  Books
  Literature reviews, systematic reviews, or meta-analyses
  Case reports or case studies.
- Study participants
  Participants are healthy individuals
- Language
  Articles not written in English

### Article Screening

**Figure 1:**
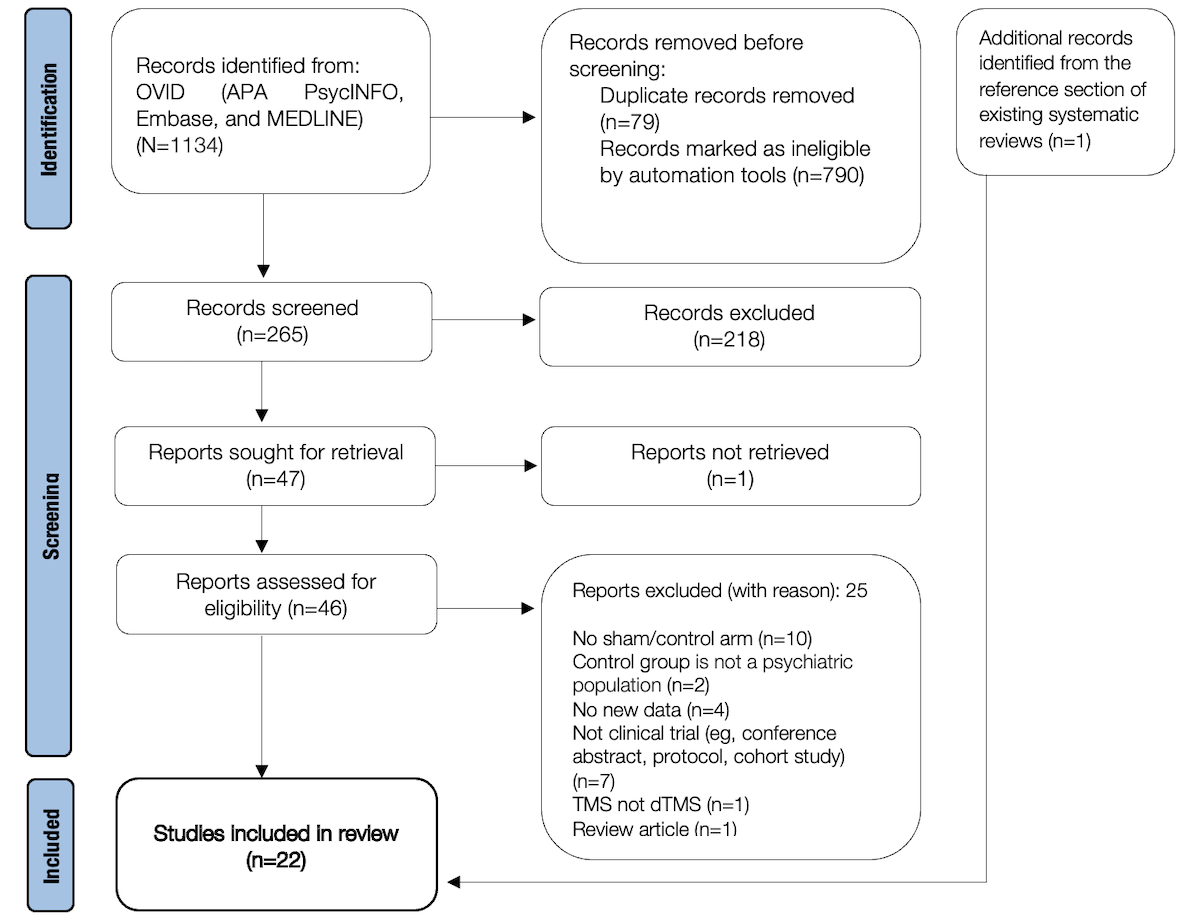
Preferred Reporting Items for Systematic Reviews and Meta-Analyses (PRISMA) flowchart. The title-abstract and full-text screenings of potentially eligible reports were performed. Articles were excluded at each stage of the screening progress according to the inclusion and exclusion criteria. dTMS: deep transcranial magnetic stimulation; TMS: transcranial magnetic stimulation.

Two authors (AD and MD) will conduct the abstract and full-text screening of relevant articles. Articles will be screened in accordance with the Preferred Reporting Items for Systematic Reviews and Meta-Analyses (PRISMA) [60] guidelines. In line with PRISMA guidelines, authors are required to report the number of records identified, included, and excluded articles (with reasons) at each stage of the screening process. PRISMA guidelines help authors ensure that potentially eligible records are being appropriately sorted and organized throughout the early systematic review process. Articles that meet the inclusion criteria upon the initial abstract screening will then become eligible for the full-text screening. Articles that meet the inclusion criteria after the full-text screening will be included in the review. The number of articles included and excluded (and the reasons for exclusion) will be reported at each stage in the screening process and will be visually represented by a PRISMA flowchart (Figure 1). The 2 reviewers (AD and MD) will consult with each other in the case article eligibility is unclear. Article eligibility discrepancies that cannot be resolved by the 2 reviewers (AD and MD) will be resolved by a third reviewer (JM).

### Risk of Bias and Quality Assessment

All articles will be assessed according to the grading of recommendations, assessment, development, and evaluations (GRADE) checklist [61,62] to evaluate the quality of data and risk of bias. Two reviewers (AD and MD) will rate articles as having a “high” or “low” risk of bias. Specifically, included articles will be assessed for selection bias, performance bias, detection bias, attrition bias, and reporting bias. Selection bias will be evaluated by identifying whether researchers used a random sequence generation and allocation concealment. Assessment of performance bias will involve determining if study personnel and participants were blinded. To calculate detection bias, reviewers will verify if studies implemented a blinding of outcome assessment. Attrition bias will be assessed by determining if at least 80% of enrolled participants were included in the final analyses (ie, comparing intention-to-treat participants to per-protocol analyses). We will also assess article quality by taking into consideration participant sample sizes and study design (ie, inclusion of control condition, baseline vs posttreatment assessment of psychiatric or cognitive symptoms) while determining the clinical efficacy of dTMS treatment.

### Data Extraction

The following data variables will be extracted from each included article: country of publication, study type, comparison type (ie, active dTMS vs sham, or standard drug therapy, or another control type), participant sample size, participant sex, and mean age. The psychiatric or cognitive disorder diagnosis and diagnostic criteria (eg, Diagnostic and Statistical Manual of Mental Disorders, 4th edition; Diagnostic and Statistical Manual of Mental Disorders, 5th edition; International Classification of Diseases, 11th edition) of patients will be recorded. Regarding treatment procedures, this study will report treatment duration, number of treatment sessions, H-coil type, and the brain regions of interest (ie, target region of stimulation). Additionally, this study will report the dTMS parameters, including frequency (Hz), intensity, pulses per session, time per session, trains per session, and intertrain interval duration. Posttreatment comparisons between dTMS efficacy in active versus sham/control groups will be reported, including data on remission rates, response rates, or posttreatment score changes on psychiatric and cognitive rating scales. Remission criteria and response criteria will also be reported. Additionally, we will include data related to side effects and adverse events. Table 2 provides an outline of the specific variables that will be extracted from studies, with respect to sample characteristics, dTMS parameters, clinical efficacy outcomes, and study design. Data from included articles will be collected and stored using Excel (Microsoft Corp) or other equivalent software. Additionally, quality assessment according to the GRADE guidelines [61,62] will be conducted for each article and recorded in a table.

The proposed systematic review will evaluate the clinical efficacy of dTMS for psychiatric and cognitive disorders by comparing active versus sham dTMS treatment groups (ie, sham-controlled trials), as well as active versus control groups (ie, without a sham coil). To do this, we will compare response rates, remission rates, and the overall efficacy of the dTMS treatment in active versus sham/control conditions. Additionally, if at least 4 substantively equivalent investigations are present in a given area, we will perform a meta-analysis to ascertain aggregate clinical effects across studies in sham/control trials. Further subgroup analyses will be conducted on age, sex, H-coil type, and dTMS parameters to the extent possible. Risk of bias and article quality assessments will be used when considering evidence and analyzing data from included studies. Moreover, the study will summarize the proposed review’s findings on the clinical efficacy of dTMS for psychiatric and (if available) cognitive disorders, identify evidence that may assist clinicians in their practice, and highlight future directions for research.

**Table 2:** Study characteristics that will be recorded from eligible articles.

Information category	Data to be extracted	
Sample characteristics	Age Gender Psychiatric or cognitive disorder diagnosis Diagnostic criteria Sample size	
Deep transcranial magnetic stimulation (dTMS) parameters	Brain region of interest (ie, location of brain stimulation) Coil type or design (ie, H-coil variety) dTMS stimulation frequency (Hz) dTMS stimulation intensity (MT%) Pulses per session Time per session (min) Trains per session; inter-train intervals	
Results: clinical efficacy	Test statistics for active versus sham/control group comparisons Test statistics for subgroup comparisons (ie, age, sex, dTMS parameter, coil design, etc) *P* values, CI values, t test values, Cohen d, Hedges g (where reported), for active vs sham/control group comparisons Adverse events Remission rates and remission criteria Response rate and response criteria Qualitative statistics for active versus sham group comparisons	
Study design	Control type (ie, sham-controlled; control activity) Comparison type (eg, active vs sham stimulation) Treatment duration Randomized versus nonrandomized Single-blind vs double-blind	

### Statistical Analysis

For the prospective meta-analysis, analyses will be conducted using Stata (StataCorp). Heterogeneity will be assessed using the *Q* test, *I*^2^, and τ^2^. A random effects model will be used, if appropriate, to account for potential between-studies variance in treatment effect.

## Results

The proposed systematic review aims to evaluate the clinical efficacy of active dTMS versus sham/control in various psychiatric (and if available) cognitive disorders. Additionally, a meta-analysis will be performed (if a sufficient number of studies are available) exploring the effects of dTMS parameters, age, sex, and H-coil type on clinical outcomes.

Thus far, 22 eligible studies have been included from the following databases: APA PsycINFO, Embase, and MEDLINE (refer to Multimedia Appendix 1). A search of the APA PsycINFO, Embase, and MEDLINE databases resulted in 1134 articles. A total of 790 articles were removed by OVID automation tools which limited articles to English, human participants, and clinical trials. Additionally, 79 duplicate records were removed by automation tools. After screening the abstracts of the remaining 265 articles, 218 were excluded. The full texts of the remaining 47 articles were then screened for eligibility. One report could not be retrieved. Following the full-text screening, 25 articles were excluded for the following reasons: (1) not having a sham or control condition (n=10); (2) the control group was not a psychiatric population (n=2); (3) no new data were reported (n=4); (4) not a clinical trial (n=7); (5) intervention was with TMS, not dTMS (n=1); and (6) being a review article (n=1). One additional article was identified from the reference section of an existing systematic review. The PubMed search did not render any additional articles.

A search of the Cochrane database still needs to be performed to identify other potential articles. The data extraction process is ongoing. Once the data extraction is complete, we will determine if there are enough suitable articles to conduct a meta-analysis. We aim to have this project complete by the end of 2023.

## Discussion

### Objectives and Limitations

The preliminary screening stages of the ongoing systematic review have rendered a total of 22 eligible articles comparing the efficacy of dTMS to a sham/control condition. While dTMS treatment has been shown to effectively alleviate clinical symptoms for psychiatric and cognitive disorders, the current state of literature lacks insight into the clinical efficacy of dTMS interventions across such disorders. Data from all 22 articles identified in the preliminary search may help to bridge this gap in the literature by conducting a comprehensive systematic review, and prospective meta-analysis, to determine the efficacy of dTMS across diverse psychiatric and cognitive disorders.

The proposed review will be the first, to our knowledge, to evaluate the clinical efficacy of dTMS across psychiatric and pertinent cognitive disorders. The knowledge gathered from this systematic review holds great clinical value. First, it can inform clinicians about the potential clinical benefits of dTMS in specific psychiatric populations. Second, by further conducting subgroup analyses (whereby a meta-analysis is deemed appropriate), this study aims to identify subgroup variables that may affect clinical outcomes following dTMS treatment. This subgroup data has valuable clinical implications, as the identification of such clinical and methodological factors may influence clinicians’ decisions to support dTMS over another treatment regimen. Thus, future dTMS treatments can be tailored to fulfill the patient’s needs and customize treatments to optimize therapeutic effects.

The systematic review that follows this protocol may have limitations. First, sham-controlled studies exploring the clinical efficacy of dTMS are limited. Second, there may not be sufficient studies to examine the clinical efficacy of dTMS for each psychiatric and cognitive disorder. Thus, a meta-analysis may not be possible for all conditions, which may hinder assessments of subgroup variables potentially influencing dTMS efficacy.

### Conclusions

In this protocol, the authors openly review the methodological procedures of a systematic review (and prospective meta-analysis) designed to effectively determine the clinical efficacy of dTMS for psychiatric and cognitive disorders. This protocol was published to ensure the originality of this research and to contribute to data availability.

## Appendix

Multimedia Appendix 1 Full search strategy and articles retrieved.

## Abbreviations

dTMS: deep transcranial magnetic stimulation
GRADE: grading of recommendations, assessment, development, and evaluations
H-coil: Hesed-coil
PRISMA: Preferred Reporting Items for Systematic Reviews and Meta-Analyses
rTMS: repetitive transcranial magnetic stimulation
TMS: transcranial magnetic stimulation

## Data Availability

The data set generated and analyzed during this study will be made available by the corresponding author upon reasonable request.
