# Supplementary material for: Clinical Efficacy of Deep Transcranial Magnetic Stimulation in Psychiatric and Cognitive Disorders: Protocol for a Systematic Review
Source: JMIR Res Protoc. 2023 May 26;12:e45213. doi: 10.2196/45213 (PMC10257110; doi:10.2196/45213)
Supplement: Multimedia Appendix 1 [file resprot_v12i1e45213_app1.doc]

**Search Strategy**

**OVID Search**

**Date of search: March 20, 2023**

Embase <1974 to 2023 March 17>

APA PsycInfo <1806 to March Week 2 2023>

Ovid MEDLINE(R) ALL <1946 to March 17, 2023>

1 dTMS.ti,ab,kw,kf. 602

2 "deep TMS".ti,ab,kw,kf. 300

3 "deep transcranial magnetic stimulation".ti,ab,kw,kf. 438

4 "deep rTMS".ti,ab,kw,kf. 156

5 "deepTMS".ti,ab,kw,kf. 5

6 "H-coil*".ti,ab,kw,kf. 316

7 "H coil*".ti,ab,kw,kf. 316

8 1 or 2 or 3 or 4 or 5 or 6 or 7 1134

9 limit 8 to english language 1114

10 limit 9 to humans 933

11 limit 10 to (adaptive clinical trial or clinical study or clinical trial, all or clinical trial, phase i or clinical trial, phase ii or clinical trial, phase iii or clinical trial, phase iv or clinical trial or controlled clinical trial or pragmatic clinical trial or randomized controlled trial) 344

12 remove duplicates from 11 265

**First screening**

Total excluded (with reasons): 218

| **Reason for exclusion** | **Number of documents** |
| --- | --- |
| Conference abstract (no results) | 60 |
| No psychiatric / cognitive disorder (e.g., Healthy participants, Parkinson’s disease, obesity, pain, chronic migraine, etc.) | 45 |
| Meta-analysis / systematic review/ review | 43 |
| Topic is unrelated | 29 |
| Case series/ case report | 12 |
| Study is not a clinical trial | 11 |
| Commentary/Response/Letter | 6 |
| Control group is healthy population | 4 |
| Duplicate article | 3 |
| No sham/control group | 3 |
| Book/chapter | 2 |
| **Total** | **218** |

INCLUDED (N= 47)

**Second screening**

| Article title | Yes or No | Reason for exclusion |
| --- | --- | --- |
| Deep rTMS of the insula and prefrontal cortex in smokers with schizophrenia: Proof-of-concept study. | Yes |  |
| Do exposure therapy processes impact the efficacy of deep TMS for obsessive-compulsive disorder? | Yes |  |
| Efficacy of Deep TMS with the H1 Coil for Anxious Depression. | No | Review |
| Repetitive Transcranial Magnetic Stimulation in Alcohol Dependence: A Randomized, Double-Blind, Sham-Controlled Proof-of-Concept Trial Targeting the Medial Prefrontal and Anterior Cingulate Cortices | Yes |  |
| Deep Transcranial Magnetic Stimulation Combined With Brief Exposure for Posttraumatic Stress Disorder: A Prospective Multisite Randomized Trial. | Yes |  |
| Repetitive transcranial magnetic stimulation in major depression: A three-arm parallel-group dose-response randomized pilot trial. | No | No sham or control group |
| A functional magnetic resonance imaging investigation of prefrontal cortex deep transcranial magnetic stimulation efficacy in adults with attention deficit/hyperactive disorder: A double blind, randomized clinical trial. | Yes |  |
| A pilot investigation of accelerated deep transcranial magnetic stimulation protocols in treatment-resistant depression. | No | No sham or control group |
| Repetitive Transcranial Magnetic Stimulation With H-Coil in Alzheimer's Disease: A Double-Blind, Placebo-Controlled Pilot Study. | yes |  |
| Efficacy and Safety of Deep Transcranial Magnetic Stimulation in Office Workers with Treatment-Resistant Depression: A Randomized, Double-Blind, Sham-Controlled Trial. | No | No access |
| Efficacy of repetitive transcranial magnetic stimulation using a figure-8-coil or an H1-Coil in treatment of major depressive disorder; A randomized clinical trial. | Yes |  |
| Efficacy and safety of deep transcranial magnetic stimulation for obsessive-compulsive disorder: A prospective multicenter randomized double-blind placebo-controlled trial. | Yes |  |
| Deep Transcranial Magnetic Stimulation Over the Medial Prefrontal and Anterior Cingulate Cortices Alters Brain Connectivity and Reduces Relapse to Alcohol Use. | No | Conference abstract of Harel et al. 2022 |
| Effects of deep transcranial magnetic stimulation over the medial PFC and ACC on relapse to alcohol use and related brain activity. | No | Conference abstract of Harel et al. 2022 |
| Efficacy and safety of repetitive transcranial magnetic stimulation using an H1-coil or figure-8-coil in the treatment of unipolar major depressive disorder: A study protocol for a randomized controlled trial | No | Protocol |
| Efficacy, tolerability, and cognitive effects of deep transcranial magnetic stimulation for late-life depression: a prospective randomized controlled trial. | Yes |  |
| Alternate day dTMS combined with SSRIs for chronic treatment resistant depression: A prospective multicenter study | No | No sham/control group |
| Randomised sham-controlled study of high-frequency bilateral deep transcranial magnetic stimulation (dTMS) to treat adult attention hyperactive disorder (ADHD): Negative results. | Yes |  |
| Cognitive outcomes of TMS treatment in bipolar depression: Safety data from a randomized controlled trial. | No | No new data |
| Deep-TMS for ADHD: A randomized sham controlled fMRI study. | No | Conference abstract |
| Deep TMS of the medial PFC and ACC for OCD: A double-blinded multi-center study | No | Conference abstract |
| Cognitive effects of deep transcranial magnetic stimulation for late-life depression. | No | Conference abstract |
| Treatment of bipolar depression with deep TMS: Results from a double-blind, randomized, parallel group, sham-controlled clinical trial. | Yes |  |
| 61% of unmedicated treatment resistant depression patients who did not respond to acute TMS treatment responded after four weeks of twice weekly deep TMS in the Brainsway pivotal trial. | No | No new data |
| Deep TMS on alcoholics: Effects on cortisolemia and dopamine pathway modulation. A pilot study | Yes |  |
| Efficacy and safety of deep transcranial magnetic stimulation for major depression: A prospective multicenter randomized controlled trial. | Yes |  |
| Double-blind, randomized sham controlled study of deep-TMS add-on treatment for negative symptoms and cognitive deficits in schizophrenia. | Yes |  |
| Smoking cessation induced by deep repetitive transcranial magnetic stimulation of the prefrontal and insular cortices: A prospective, randomized controlled trial. | Yes |  |
| Effectiveness of deep transcranial magnetic stimulation combined with a brief exposure procedure in post-traumatic stress disorder-a pilot study. | Yes |  |
| Deep transcranial magnetic stimulation for the treatment of auditory hallucinations: A preliminary open-label study. | No | No sham/control group |
| Deep transcranial magnetic stimulation over the prefrontal cortex: Evaluation of antidepressant and cognitive effects in depressive patients. | No | Control group is not psychiatric population |
| Long-term outcomes of a course of deep TMS for treatment-resistant OCD | Yes |  |
| Modifications of cognitive performance in the Stroop task following deep rTMS treatment course in OCD patients. | No | No new data |
| Deep transcranial magnetic stimulation for obsessive-compulsive disorder is efficacious even in patients who failed multiple medications and CBT | No | No new data |
| Deep transcranial magnetic stimulation for the treatment of negative symptoms in schizophrenia: Beyond an antidepressant effect | No | No sham/control group |
| Clinical and electrophysiological outcomes of deep TMS over the medial prefrontal and anterior cingulate cortices in OCD patients. | Yes |  |
| Factors associated with response after deep transcranial magnetic stimulation in a real-world clinical setting: Results from the first 40 cases of treatment-resistant depression. | No | Not clinical trial (cohort study) |
| Efficacy, safety and tolerability of augmentative rTMS in treatment of major depressive disorder (MDD): A prospective cohort study in Croatia | No | TMS not dTMS |
| Deep transcranial magnetic stimulation of the dorsolateral prefrontal cortex in alcohol use disorder patients: Effects on dopamine transporter availability and alcohol intake | Yes |  |
| Antidepressant effectiveness of deep Transcranial Magnetic Stimulation (dTMS) in patients with Major Depressive Disorder (MDD) with or without Alcohol Use Disorders (AUDs): A 6-month, open label, follow-up study. | No | No sham/control group |
| Add-on deep transcranial magnetic stimulation (dTMS) in patients with dysthymic disorder comorbid with alcohol use disorder: A comparison with standard treatment. | Yes |  |
| H-coil repetitive transcranial magnetic stimulation for treatment major depressive disorder: An 18-week continuation safety and feasibility study. | No | No sham/control group |
| Deep transcranial magnetic stimulation add-on for the treatment of auditory hallucinations: A double-blind study | Yes |  |
| Deep transcranial magnetic stimulation add-on for treatment of negative symptoms and cognitive deficits of schizophrenia: A feasibility study | No | No sham/control group |
| H-Coil repetitive transcranial magnetic stimulation for the treatment of bipolar depression: An add-on, safety and feasibility study | No | Control group is not psychiatric population |
| Deep TMS in a resistant major depressive disorder: A brief report. | No | No sham or control group |
| Response to deep TMS in depressive patients with previous electroconvulsive treatment. | No | No sham or control group |

Total included articles (N= 21)

| 1. Moeller SJ, Gil R, Weinstein JJ, Baumvoll T, Wengler K, Fallon N, Van Snellenberg JX, Abeykoon S, Perlman G, Williams J, Manu L, Slifstein M, Cassidy CM, Martinez DM, Abi-Dargham A. Deep rTMS of the insula and prefrontal cortex in smokers with schizophrenia: Proof-of-concept study. Schizophrenia (Heidelb). 2022 Feb 25;8(1):6. doi: 10.1038/s41537-022-00224-0. PMID: 35217662; PMCID: PMC8881463. 2. Guzick, A. G., Schweissing, E., Tendler, A., Sheth, S. A., Goodman, W. K., & Storch, E. A. (2022). Do exposure therapy processes impact the efficacy of deep TMS for obsessive-compulsive disorder? *Journal of Obsessive-Compulsive and Related Disorders*, *35*, 100756. https://doi.org/10.1016/j.jocrd.2022.100756 3. Harel M, Perini I, Kämpe R, Alyagon U, Shalev H, Besser I, Sommer WH, Heilig M, Zangen A. Repetitive Transcranial Magnetic Stimulation in Alcohol Dependence: A Randomized, Double-Blind, Sham-Controlled Proof-of-Concept Trial Targeting the Medial Prefrontal and Anterior Cingulate Cortices. Biol Psychiatry. 2022 Jun 15;91(12):1061-1069. doi: 10.1016/j.biopsych.2021.11.020. Epub 2021 Dec 6. PMID: 35067356. 4. Isserles M, Tendler A, Roth Y, Bystritsky A, Blumberger DM, Ward H, Feifel D, Viner L, Duffy W, Zohar J, Keller CJ, Bhati MT, Etkin A, George MS, Filipcic I, Lapidus K, Casuto L, Vaishnavi S, Stein A, Deutsch L, Deutsch F, Morales O, Daskalakis ZJ, Zangen A, Ressler KJ. Deep Transcranial Magnetic Stimulation Combined With Brief Exposure for Posttraumatic Stress Disorder: A Prospective Multisite Randomized Trial. Biol Psychiatry. 2021 Nov 15;90(10):721-728. doi: 10.1016/j.biopsych.2021.04.019. Epub 2021 May 4. PMID: 34274108. 5. Bleich-Cohen M, Gurevitch G, Carmi N, Medvedovsky M, Bregman N, Nevler N, Elman K, Ginou A, Zangen A, Ash EL. A functional magnetic resonance imaging investigation of prefrontal cortex deep transcranial magnetic stimulation efficacy in adults with attention deficit/hyperactive disorder: A double blind, randomized clinical trial. Neuroimage Clin. 2021;30:102670. doi: 10.1016/j.nicl.2021.102670. Epub 2021 Apr 18. PMID: 34215144; PMCID: PMC8102620. 6. Leocani L, Dalla Costa G, Coppi E, Santangelo R, Pisa M, Ferrari L, Bernasconi MP, Falautano M, Zangen A, Magnani G, Comi G. Repetitive Transcranial Magnetic Stimulation With H-Coil in Alzheimer's Disease: A Double-Blind, Placebo-Controlled Pilot Study. Front Neurol. 2021 Feb 18;11:614351. doi: 10.3389/fneur.2020.614351. PMID: 33679572; PMCID: PMC7930223.  Filipčić I, Šimunović Filipčić I, Milovac Ž, Sučić S, Gajšak T, Ivezić E, Bašić S, Bajić Ž, Heilig M. Efficacy of repetitive transcranial magnetic stimulation using a figure-8-coil or an H1-Coil in treatment of major depressive disorder; A randomized clinical trial. J Psychiatr Res. 2019 Jul;114:113-119. doi: 10.1016/j.jpsychires.2019.04.020. Epub 2019 Apr 26. PMID: 31059991.  1. Carmi L, Tendler A, Bystritsky A, Hollander E, Blumberger DM, Daskalakis J, Ward H, Lapidus K, Goodman W, Casuto L, Feifel D, Barnea-Ygael N, Roth Y, Zangen A, Zohar J. Efficacy and Safety of Deep Transcranial Magnetic Stimulation for Obsessive-Compulsive Disorder: A Prospective Multicenter Randomized Double-Blind Placebo-Controlled Trial. Am J Psychiatry. 2019 Nov 1;176(11):931-938. doi: 10.1176/appi.ajp.2019.18101180. Epub 2019 May 21. PMID: 31109199.  Kaster TS, Daskalakis ZJ, Noda Y, Knyahnytska Y, Downar J, Rajji TK, Levkovitz Y, Zangen A, Butters MA, Mulsant BH, Blumberger DM. Efficacy, tolerability, and cognitive effects of deep transcranial magnetic stimulation for late-life depression: a prospective randomized controlled trial. Neuropsychopharmacology. 2018 Oct;43(11):2231-2238. doi: 10.1038/s41386-018-0121-x. Epub 2018 Jun 18. PMID: 29946106; PMCID: PMC6135812.Paz Y, Friedwald K, Levkovitz Y, Zangen A, Alyagon U, Nitzan U, Segev A, Maoz H, Koubi M, Bloch Y. Randomised sham-controlled study of high-frequency bilateral deep transcranial magnetic stimulation (dTMS) to treat adult attention hyperactive disorder (ADHD): Negative results. World J Biol Psychiatry. 2018 Oct;19(7):561-566. doi: 10.1080/15622975.2017.1282170. Epub 2017 Jan 31. PMID: 28090806.  1. Tavares DF, Myczkowski ML, Alberto RL, Valiengo L, Rios RM, Gordon P, de Sampaio-Junior B, Klein I, Mansur CG, Marcolin MA, Lafer B, Moreno RA, Gattaz W, Daskalakis ZJ, Brunoni AR. Treatment of Bipolar Depression with Deep TMS: Results from a Double-Blind, Randomized, Parallel Group, Sham-Controlled Clinical Trial. Neuropsychopharmacology. 2017 Dec;42(13):2593-2601. doi: 10.1038/npp.2017.26. Epub 2017 Feb 1. PMID: 28145409; PMCID: PMC5686495. 2. Ceccanti M, Inghilleri M, Attilia ML, Raccah R, Fiore M, Zangen A, Ceccanti M. Deep TMS on alcoholics: effects on cortisolemia and dopamine pathway modulation. A pilot study. Can J Physiol Pharmacol. 2015 Apr;93(4):283-90. doi: 10.1139/cjpp-2014-0188. Epub 2015 Mar 2. PMID: 25730614. 3. Levkovitz Y, Isserles M, Padberg F, Lisanby SH, Bystritsky A, Xia G, Tendler A, Daskalakis ZJ, Winston JL, Dannon P, Hafez HM, Reti IM, Morales OG, Schlaepfer TE, Hollander E, Berman JA, Husain MM, Sofer U, Stein A, Adler S, Deutsch L, Deutsch F, Roth Y, George MS, Zangen A. Efficacy and safety of deep transcranial magnetic stimulation for major depression: a prospective multicenter randomized controlled trial. World Psychiatry. 2015 Feb;14(1):64-73. doi: 10.1002/wps.20199. PMID: 25655160; PMCID: PMC4329899. 4. Rabany L, Deutsch L, Levkovitz Y. Double-blind, randomized sham controlled study of deep-TMS add-on treatment for negative symptoms and cognitive deficits in schizophrenia. J Psychopharmacol. 2014 Jul;28(7):686-90. doi: 10.1177/0269881114533600. Epub 2014 May 14. PMID: 24829210. 5. Dinur-Klein L, Dannon P, Hadar A, Rosenberg O, Roth Y, Kotler M, Zangen A. Smoking cessation induced by deep repetitive transcranial magnetic stimulation of the prefrontal and insular cortices: a prospective, randomized controlled trial. Biol Psychiatry. 2014 Nov 1;76(9):742-9. doi: 10.1016/j.biopsych.2014.05.020. Epub 2014 Jun 5. PMID: 25038985. 6. Isserles M, Shalev AY, Roth Y, Peri T, Kutz I, Zlotnick E, Zangen A. Effectiveness of deep transcranial magnetic stimulation combined with a brief exposure procedure in post-traumatic stress disorder--a pilot study. Brain Stimul. 2013 May;6(3):377-83. doi: 10.1016/j.brs.2012.07.008. Epub 2012 Aug 18. PMID: 22921765. 7. Harmelech T, Tendler A, Arikan MK, Çetin HL, Esmeray MT, Ilhan R, Vidrine R, Muir O, MacMillan C, Sinclair R, Shakir S, Kent D, Evangelidis N, Roth Y. Long-term outcomes of a course of deep TMS for treatment-resistant OCD. Brain Stimul. 2022 Jan-Feb;15(1):226-228. doi: 10.1016/j.brs.2021.12.011. Epub 2022 Jan 1. PMID: 34982981. 8. Carmi L, Alyagon U, Barnea-Ygael N, Zohar J, Dar R, Zangen A. Clinical and electrophysiological outcomes of deep TMS over the medial prefrontal and anterior cingulate cortices in OCD patients. Brain Stimul. 2018 Jan-Feb;11(1):158-165. doi: 10.1016/j.brs.2017.09.004. Epub 2017 Sep 6. PMID: 28927961. 9. Addolorato G, Antonelli M, Cocciolillo F, Vassallo GA, Tarli C, Sestito L, Mirijello A, Ferrulli A, Pizzuto DA, Camardese G, Miceli A, Diana M, Giordano A, Gasbarrini A, Di Giuda D. Deep Transcranial Magnetic Stimulation of the Dorsolateral Prefrontal Cortex in Alcohol Use Disorder Patients: Effects on Dopamine Transporter Availability and Alcohol Intake. Eur Neuropsychopharmacol. 2017 May;27(5):450-461. doi: 10.1016/j.euroneuro.2017.03.008. Epub 2017 Apr 6. PMID: 28390775. 10. Girardi P, Rapinesi C, Chiarotti F, Kotzalidis GD, Piacentino D, Serata D, Del Casale A, Scatena P, Mascioli F, Raccah RN, Brugnoli R, Digiacomantonio V, Ferri VR, Ferracuti S, Zangen A, Angeletti G. Add-on deep transcranial magnetic stimulation (dTMS) in patients with dysthymic disorder comorbid with alcohol use disorder: a comparison with standard treatment. World J Biol Psychiatry. 2015 Jan;16(1):66-73. doi: 10.3109/15622975.2014.925583. Epub 2014 Aug 20. PMID: 25140585. 11. Rosenberg O, Gersner R, Klein LD, Kotler M, Zangen A, Dannon P. Deep transcranial magnetic stimulation add-on for the treatment of auditory hallucinations: a double-blind study. Ann Gen Psychiatry. 2012 May 6;11:13. doi: 10.1186/1744-859X-11-13. PMID: 22559192; PMCID: PMC3355036. |
| --- |

**PubMed search**

**Date of search: March 23, 2023**

Search keywords: ("deep TMS") OR ("deep rTMS") OR ("deep transcranial magnetic stimulation") OR ("H coil") OR ("H-coil") OR (dTMS) OR (deepTMS)

No date limits applied

Filters applied: Limit article type to *clinical trial* or *randomized controlled trial*

Articles retrieved: 58

Additional (not found in OVID search) articles retrieved from PubMed Search = 0

**Manual search (references of other meta-analyses & systematic reviews)**

1. Martinez D, Urban N, Grassetti A, Chang D, Hu MC, Zangen A, Levin FR, Foltin R, Nunes EV. Transcranial Magnetic Stimulation of Medial Prefrontal and Cingulate Cortices Reduces Cocaine Self-Administration: A Pilot Study. Front Psychiatry. 2018 Mar 16;9:80. doi: 10.3389/fpsyt.2018.00080. PMID: 29615935; PMCID: PMC5864905.

**Retrieved from**: Zhang JJQ, Fong KNK, Ouyang RG, Siu AMH, Kranz GS. Effects of repetitive transcranial magnetic stimulation (rTMS) on craving and substance consumption in patients with substance dependence: a systematic review and meta-analysis. Addiction. 2019 Dec;114(12):2137-2149. doi: 10.1111/add.14753. Epub 2019 Aug 16. PMID: 31328353.
